# Supplementary figures and images for: Strain and temperature dependent aggregation of Candida auris is attenuated by inhibition of surface amyloid proteins
Source: Cell Surf. 2023 Jul 24;10:100110. doi: 10.1016/j.tcsw.2023.100110 (PMC10407437; doi:10.1016/j.tcsw.2023.100110)

A)

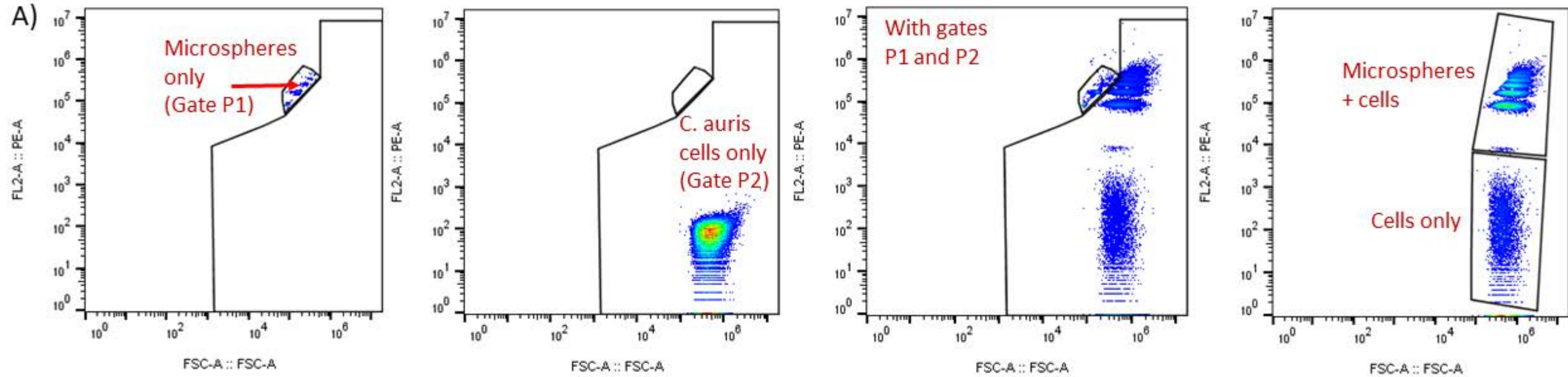

B)

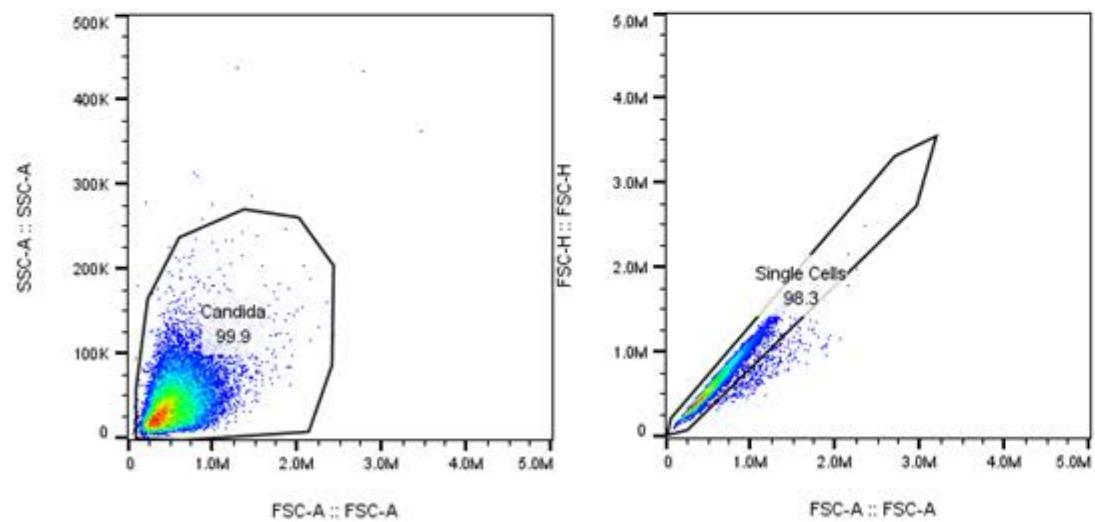

Supplement: Supplementary data 4 — Gating strategies. A) Gating strategy for adhesion assay. Gate P1 represents uncoated carboxylated yellow-green fluorescent polystyrene microspheres only, Gate P2 represents C. auris cells without polystyrene microspheres. Representative density plot (far right) shows systematic identification of C. auris cells that have adhered to polystyrene microspheres. B) Gating strategy for cell wall staining. Representative density plots showing whole population (left) of C. auris cells using side scatter area on y-axis and forward scatter area on x-axis. Single-cells population (right) identified using forward scatter height on y-axis and forward scatter area on x-axis. [file mmc4.pdf]
